# Interindividual variability in gut microbial formation of the hop phytoestrogen 8-prenylnaringenin results in elevated but sub-toxic internal exposures

**DOI:** 10.1007/s00204-026-04329-8

**Published:** 2026-03-10

**Authors:** Maja Stevanoska, Michelle Cremona, Karsten Beekmann, Shana J. Sturla, Georg Aichinger

**Affiliations:** 1 Laboratory of Toxicology, Department of Health Sciences and Technology, ETH Zurich, Schmelzbergstrasse 9, LFO D15.2, 8092 Zurich, Switzerland; 2 Wageningen Food Safety Research (WFSR), Wageningen, The Netherlands

**Keywords:** Microbial metabolism, Pharmacokinetics, PBK modeling, Biotransformation, Hop polyphenols, Estrogenicity

## Abstract

The gut microbiome converts the prenylated polyphenol isoxanthohumol (iXN), a natural constituent of hops found in beer, into 8-prenylnaringenin (8-PN), a potent phytoestrogen associated with endocrine-disrupting effects. Following oral exposure, interindividual differences in microbiome composition may lead to variable systemic 8-PN concentrations and consequently to differences in susceptibility to toxicity. To characterize the contribution of gut microbiota to health effects of hop polyphenols, a human physiologically based kinetic (PBK) model that includes microbial 8-PN formation was developed. Ex vivo fecal fermentation coupled to LC–MS/MS revealed substantial interindividual variation in biotransformation capacity. Derived kinetic parameters were incorporated into the PBK model, which was subsequently used to predict systemic 8-PN exposure while accounting for interindividual variability. Model simulations indicated that high iXN metabolizers experience approximately two-fold more internal 8-PN exposure than low metabolizers. Estrogenicity of the predicted uterine 8-PN concentrations was assessed via alkaline phosphatase induction in Ishikawa cells. Even in high metabolizers, systemic 8-PN concentrations appeared to remain below levels of concern regarding endocrine disruption. These findings highlight the importance of accounting for interindividual variability in gut microbial biotransformation when predicting xenobiotic toxicokinetics and illustrate the applicability of microbiome-competent PBK modeling for predicting the systemic fate of gut microbial metabolites.

**Supplementary Information:**

The online version contains supplementary material available at https://doi.org/10.1007/s00204-026-04329-8.

## Introduction

The gut microbiome metabolizes xenobiotics, including dietary compounds, thereby producing metabolites with altered bioactivities, potentially including toxicity to the consumer (Koppel et al. 2017; Zimmermann et al. 2019). However, microbiome-mediated chemical metabolism is often insufficiently addressed in current chemical safety assessment (Ampatzoglou et al. 2022). The latter is largely based on animal testing, thus raising ethical concerns, requiring substantial resources, and introducing uncertainty due to interspecies differences in response to chemical exposure, including microbiome-induced variation. Consequently, there is a shift towards new approach methodologies that combine in vitro and in silico techniques (Krewski et al. 2020), wherein physiologically based kinetic (PBK) models are used to predict the systemic fate of chemicals in humans. By integrating the gut microbiome as a metabolic compartment in PBK models, systemic levels of microbial metabolites can be predicted, thereby expanding the capacity to define the bioactivity or toxicity of xenobiotics metabolized by the microbiome and their biotransformation products (Stevanoska et al. 2024).

**Fig. 1:**
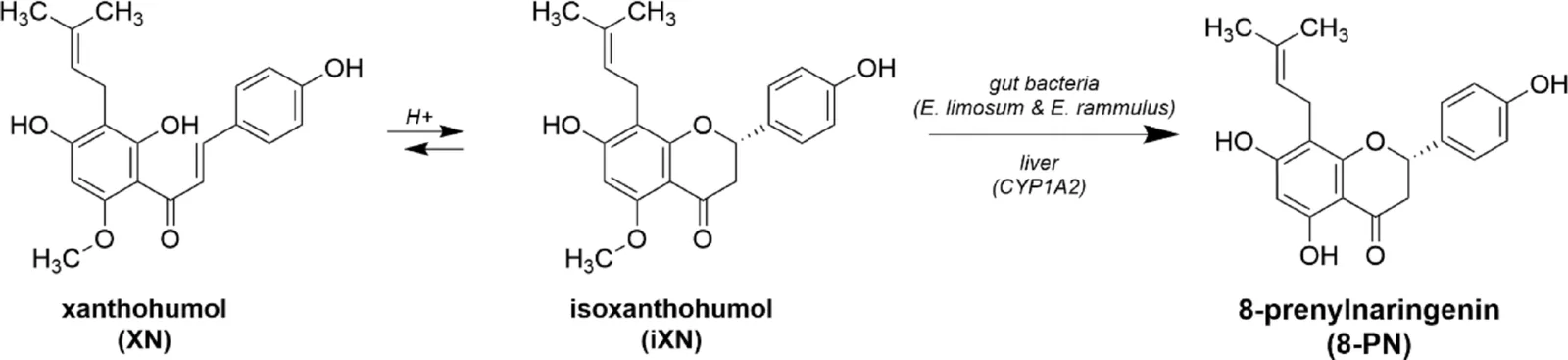
Xanthohumol (XN) undergoes acid- or heat-induced cyclization to isoxanthohumol (iXN), which is subsequently biotransformed into 8-prenylnaringenin (8-PN) by the hepatic enzyme CYP1A2 and by the gut microbial strains *E. limosum* and *E. rammulus*

Interactions of microbial metabolites with human hormone receptors may lead to endocrine disruption and related adverse health effects (Aguilera et al. 2020). For example, prenylated polyphenols, naturally abundant in hops, can be converted by the gut microbiome into one of the most potent phytoestrogens known, namely 8-prenylnaringenin (8-PN) (Possemiers et al. 2005). Several studies have shown the dual nature of 8-PN, some showing beneficial effects such as alleviating menopausal symptoms or improving bone health, while others describe 8-PN as a potential endocrine disruptor (Pohjanvirta and Nasri 2022). Hop polyphenol exposure mainly results from drinking beer and hopped beverages. Additionally, hop supplements are used in alternative medicine practices by women to relieve post-menopausal symptoms (Keiler et al. 2013). In hops, the phytoestrogen 8-PN is present in low concentrations, while the major hop polyphenols are the chalcone xanthohumol (XN) and the flavonoid isoxanthohumol (iXN) (Stevens 1999). During the brewing process and also upon acidic cyclization in the stomach following ingestion, XN is converted to iXN, which is the precursor to 8-PN (Nikolic et al. 2005). Additionally, iXN is converted to 8-PN by CYP1A2-catalyzed *O*-demethylation in the liver (Guo et al. 2006) and by gut-resident bacteria such as Eubacterium *limosum* and E. *rammulus* (Fig. 1) (Moens et al. 2020; Paraiso et al. 2019). This bioactivation could be relevant to host toxicity; however, the extent to which the gut microbiome contributes to this metabolic process remains unknown. Systemic levels of iXN and 8-PN in blood and tissues of toxicological concern were in the picomolar range as predicted by a PBK model for both compounds. However, the potential gut microbial formation of 8-PN was not addressed (Stevanoska et al. 2025).

In this study, we evaluated the potential role of gut microbial metabolism in endocrine-related toxicity of hop polyphenols by measuring the kinetics of microbial 8-PN formation and then extending the prior PBK model by integrating the gut microbiome. The microbiome-competent PBK model included interindividual differences in the microbiome’s metabolic capacities to form 8-PN. The model was subsequently used to predict uterine iXN and 8-PN concentrations after beer intake, which were compared with estrogenicity data from alkaline phosphatase (ALP) cell-based assays to support a preliminary evaluation of potential endocrine-disrupting effects.

## Methods

### Chemicals and reagents

iXN (primary reference standard, purity ≥ 90.0%), 8-PN (analytical standard, purity ≥ 95.0%), glycerol, charcoal-stripped fetal bovine serum (FBS), fulvestrant (purity > 98%), 17β-estradiol (purity ≥ 98%), ethyl acetate, 4-nitrophenyl phosphate (purity ≥ 97%), diethanolamine, Triton-X, sodium dodecyl sulfate, dimethylformamide and MTT were purchased from Sigma–Aldrich (Buchs, Switzerland). DMSO was purchased from VWR (Dietikon, Switzerland), and MS-grade water was purchased from Merck-Millipore (Canada). Dulbecco’s Modified Eagle Medium (DMEM), phenol red-free DMEM, FBS, and penicillin/streptomycin were purchased from Gibco, Life Technologies Limited (Paisley, UK). Urolithin A (purity ≥ 95.0%) was purchased from abcr. Acetonitrile, methanol (MS-grade), and formic acid were purchased from Fisher Chemical. Glacial acetic acid and magnesium dichloride were purchased from Merck (Switzerland). MacFarlane media ingredients and corresponding vendors can be found in the supplementary information (Table 1).

### PBK model development and parameterization

A PBK model was adapted from a previously published model for hop polyphenols that was evaluated by comparison to human pharmacokinetic data for iXN and 8-PN (Stevanoska et al. 2025). Briefly, the model contained separate compartments for blood, adipose, liver, gut, uterus, and kidney tissue, while the remaining organs were grouped as slowly perfused tissues (bone, skin, and muscle) and rapidly perfused tissues (heart, brain, and lungs). Standard physiological metrics for an average female with a body weight of 60 kg (Valentin 2002), including relative tissue volumes, blood flow rates, gastrointestinal transit times, and glomerular filtration rate, were used to parameterize the model. Compound-specific physicochemical parameters were derived from LogP and pKa values for iXN and its metabolites using the QIVIVE toolbox to calculate tissue partition coefficients and unbound plasma fractions (Punt et al. 2021). Additionally, the kinetic parameters for the microbial formation of 8-PN were included as described below. Besides the microbial formation of 8-PN, the hepatic conversion of iXN to 8-PN by CYPA12 was included as previously described (Stevanoska et al. 2025). Briefly, it was scaled using the intersystem extrapolation factor, the reported abundance of CYP1A2 (pmol/mg protein), and the microsomal protein content relative to liver weight (Chen et al. 2011). The new microbiome-competent PBK model was applied to predict iXN and 8-PN tissue concentrations corresponding to daily intake of one beer with varying iXN and 8-PN content, over a seven-day period. The specific amounts of iXN and 8-PN in the beers used as the intake dose can be found in the supplementary information (Table 2). Additionally, a sensitivity analysis was performed to assess the physiological parameters influencing the predicted blood concentration of 8-PN. The sensitivity analysis was performed using parameters either for pooled or individual microbial kinetics of 8-PN formation. To assess the sensitivity, each parameter was increased by 5%, and normalized sensitivity coefficients (SC) were calculated using the formula *SC=(C′−C) ∕ (P′−P)×P ∕ C* (Evans and Andersen 2000), where C and C’ refer to the baseline or changed blood C_max_ of 8-PN, and P and P’ refer to the corresponding baseline and increased parameter value, respectively. To account for variability and uncertainty in the apparent most influential model parameters (supplementary information Table 3), Monte Carlo simulations (*n* = 1000) were performed. Parameter distributions were assigned as normal or log-normal and constrained to physiologically plausible human values. For normal distributions, SD was calculated as $\:S=\mu\:\times\:CV$ with CV set to 30% for physiological parameters, 20% for surface areas and partition coefficients, and 70% for enzyme kinetic parameters based on Kang et al. (Kang et al. 2024). For log-normal distributions, log-space variability was defined as $\:{\sigma\:}_{ln}=\sqrt{\mathrm{l}\mathrm{n}(1+{CV}^{2})}$, and the SD was computed as $\:S=\mu\:\times\:\sqrt{{e}_{ln}^{\sigma\:2}-1}$. Physiological constraints were applied to ensure that parameters such as body weight, small intestine surface area, and large intestine surface area remained within biologically plausible ranges.

### Human stool sample collection and processing

Stool samples were collected from 12 individuals with written informed consent and confirmation of inclusion criteria compliance. Donor criteria included having regular bowel movements ranging from once every three days to three times per day, no chronic inflammatory bowel conditions, no use of immunosuppressants, blood thinners, or medications affecting gut transit/digestion within the month before sample donation, absence of regular intestinal discomfort, and no history of operative intestinal interventions. The sample collection was anonymous, and the researchers were not able to link the sample number with its respective donors. Each donor provided a one-time donation in a container with an AnaeroGen™ bag for the generation of an anaerobic atmosphere. Following the collection, the samples were processed immediately in an anaerobic chamber (5% H_2_, 10% CO_2,_ and 85% N_2_). A fecal slurry was prepared by diluting 25% w/v in 15% anaerobic glycerol in PBS. The samples were aliquoted and stored at −80 °C until further use. This study was exempted from review by the Cantonal Ethics Commission of Zurich.

### Determination of model parameters for microbial metabolism

To determine kinetic parameters (V_max_ and K_m_) of the microbial biotransformation of iXN to 8-PN, fecal slurries were incubated in triplicate with iXN (1–55 µM) under humified anaerobic conditions at 37 °C. Preliminary assays were performed to identify suitable time points to monitor 8-PN formation (0, 1, 3, 6, 24 h); 8-PN was detected at 24 h. All stool samples were analyzed for background levels of iXN and 8-PN to exclude contributions from recent beer or hops-containing product consumption by the donor. No detectable levels of either compound were found. Fecal slurries from all 12 donors were pooled and diluted in a 1:1,5 ratio with MacFarlane media, resulting in 10% pooled fecal slurry. The slurries were spiked with iXN dissolved in DMSO (final DMSO concentration was 0.1%) or DMSO as a solvent control. The 24-well plates were incubated for 24 h, and samples were removed at time points 0, 6, and 24 h. After incubation, urolithin A was added as an internal standard to correct for potential material losses during sample preparation. Subsequently, the samples were extracted by adding ethyl acetate with 0.1% formic acid and centrifuged at 19,083 × g for 10 min at room temperature. Supernatants were collected and completely dried using a miVac SpeedVac and reconstituted in 70% methanol. The samples were stored at −20 °C until further use and diluted 1:10 in 70% methanol before analysis. All analyses were performed using an Orbitrap iDX (Thermo Fisher Scientific) LC–MS/MS system. Briefly, analytes were separated on a Phenomenex Synergi™ 4 μm Polar-RP column (80 Å, 30 × 2 mm). The temperature of the column compartment was maintained at 40 °C, while the samples in the autosampler were maintained at 4 °C. An injection volume of 5 µL was used. The mobile phase consisted of LC–MS grade water (solvent A) and acetonitrile (solvent B), each containing 0.1% formic acid. The flow was adjusted to 0.4 mL/min with a gradient profile starting at 5% solvent B for the first 3 min, transitioning to 100% solvent B by 4 min, and returning to 5% solvent B from 4.1 to 5.5 min. The limit of quantification of iXN and 8-PN was 1 nM. The microbial conversion of iXN to 8-PN in human stool samples followed Michaelis-Menten kinetics, and concentration-dependent metabolite formation was fitted using GraphPad Prism 10 to the Michaelis-Menten equation:

$$\:v={V}_{max}*\left[S\right]t/({K}_{m}+[S\left]\right)$$

V_max_ was expressed in µmol/h/g of feces and scaled to the whole body on the basis of gastrointestinal tract contents (Wang et al. 2022).

### Assessment of 8-PN formation in individual stool samples

To assess interindividual differences in microbial 8-PN formation, each of the twelve stool samples used in the pooled fecal slurry was evaluated individually for its capacity to transform iXN to 8-PN. Samples were diluted with MacFarlane media (1:1.5, v/v) and incubated with 10 µM of iXN as a substrate or with DMSO as a control (24 h, 37 °C). Thereafter, samples were processed and analyzed by LC–MS/MS as described above for the pooled fecal slurry samples. The results were normalized to the average amount of 8-PN formed from four independent anaerobic fermentations in pooled fecal slurry at 10 µM iXN. The 8-PN concentrations measured for individual donors were divided by the corresponding pooled slurry result, and this ratio was used to scale the pooled slurry V_max_ and estimate donor-specific V_max_.

### Cell culture

Ishikawa cells, a human endometrial adenocarcinoma cell line, established from a 39-year-old Japanese woman in 1980 (Nishida 2002) were purchased from the European Collection of Authenticated Cell Cultures and cultured in DMEM 10% (v/v) fetal bovine serum and 1% (v/v) penicillin/streptomycin. The cells were incubated in a humidified atmosphere (5% CO_2_, 37 °C ). Cells were sub-cultivated twice a week and used for experiments up to passage 32, in which logarithmic growth was maintained, and results were reproducible amongst replicate measurements.

### Alkaline phosphatase (ALP) assay

To measure the estrogenicity of iXN and 8-PN, ALP assays were performed as previously described (Aichinger et al. 2021). Briefly, 15’000 Ishikawa cells per well were seeded in 96-well plates and allowed to attach for 24 h. Before exposing the cells to the compounds of interest, DMEM was replaced with phenol red-free DMEM supplemented with 10% (v/v) charcoal-stripped FBS and 1% (v/v) P/S (assay medium). iXN and 8-PN were dissolved in DMSO and subsequently diluted with assay medium to the desired concentrations, ensuring a final DMSO concentration of ≤ 0.1%. Cells were exposed for 48 h to varying concentrations of 8-PN and iXN, alone or in combination, 1 nM 17β-estradiol (E2) as a positive control, or to DMSO (0.1%) as a solvent control. Additionally, co-incubation with the estrogen receptor antagonist, fulvestrant, was used to confirm that the observed effects were attributed to the test compounds interacting with ER. Cells were washed three times with PBS and lysed by shock freezing at −80 °C. After thawing for 5 min at room temperature, detection buffer (5 mM 4-nitrophenyl phosphate, 1 M diethanolamine, 0.24 mM magnesium dichloride, pH 9.8) was added, and incubated for 10 min. The conversion of 4-nitrophenyl phosphate to 4-nitrophenol was monitored at 405 nm for 1 h every 3 min intervals using an Infinite Pro M200 Tecan plate reader. ER-dependent induction of ALP was quantified from the slope of the dose-response curves with values relative to the solvent control (0%) and positive control (1nM E2, 100%).

### MTT cell viability assay

To exclude cytotoxicity as a cause of ALP responses, cell viability was assessed in parallel. Ishikawa cells were seeded and exposed to the same treatments as in the ALP assay, with 0.1% DMSO as a negative control and 0.1% Triton-X applied after 48 h as a positive control. Following 1 h incubation with Triton-X, the culture medium was removed, and 0.45 mg/mL MTT solution was added. After 2 h, formazan crystals were solubilized by adding 40% (v/v) dimethylformamide, 2% (v/v) glacial acetic acid, and 16% (w/v) sodium dodecyl sulfate, and absorbance was measured at 570 nm. Values were normalized to the negative control (100%) and the positive control (0%).

### Combination index analysis

To evaluate potential interactions between 8-PN and iXN in estrogenicity, the combination index (CI) was calculated using the Chou-Talalay method (Chou 2006). In brief, this approach applies the median-effect principle to assess synergistic, additive, or antagonistic interactions by integrating dose-response data of single compounds and their combinations. Normalized ALP activity was used to calculate the fraction affected ($\:{f}_{a}$), defined as the observed effect relative to the maximum of the positive control (1 nM E2). To account for the logarithmic dose-effect relationship, a log-transformed dose-response equation was used to determine the effective doses ($\:{D}_{x}$) for each compound, calculated as: $\:{D}_{x}=\:{10}^{\left(\frac{\mathrm{log}\left(\frac{fa}{fu}\right)-b}{m}\right)}$ where $\:{f}_{u\:}=1-\:{f}_{a},\:b=-m*\mathrm{l}\mathrm{o}\mathrm{g}\:\left({D}_{m}\right)$, and $\:{D}_{m}$ is the median-effect dose. The CI for the fixed-ratio combinations was calculated as: $\:CI=\frac{{D}_{1}}{{D}_{x}^{\left(1\right)}}+\frac{{D}_{2}}{{D}_{x}^{\left(2\right)}}\:$ where $\:{D}_{1}$ and $\:{D}_{2}$ are the doses of 8-PN and iXN in the combination. The * CI* values were interpreted as follows: * CI<1* indicates synergism, * CI=1* indicates additivity, and * CI>1* indicates antagonism.

## Results

### Kinetic parameters of 8-PN formation in pooled human stool samples

**Fig. 2:**
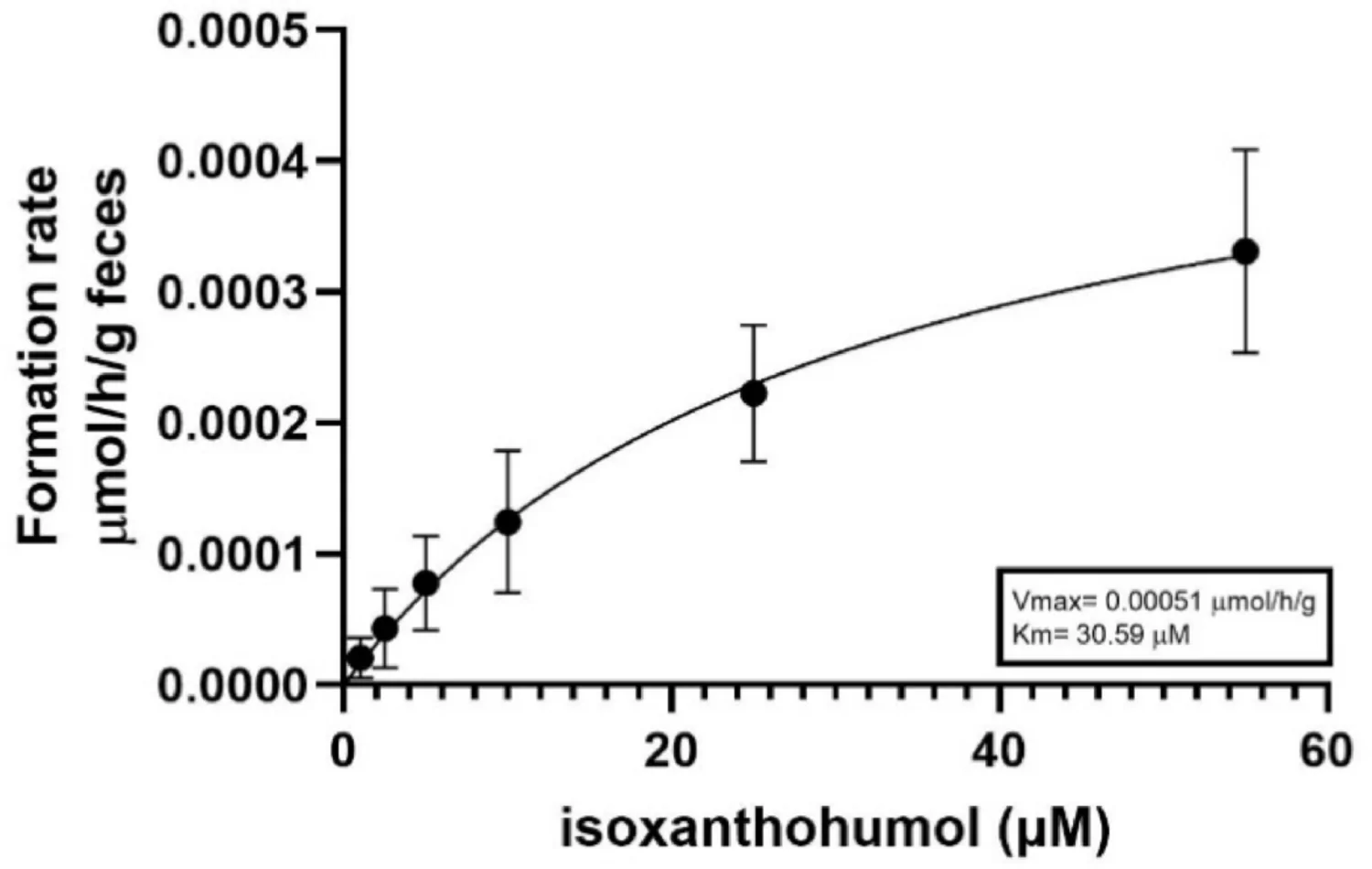
Kinetic analysis of microbial 8-PN formation from iXN as a substrate in pooled fecal slurries under anaerobic conditions. The relationship of iXN concentration (x-axis) with 8-PN formation rate (y-axis) is depicted with Michaelis-Menten curve fitting. Data are presented as mean ± SD of at least three independent experiments

To characterize the kinetics of 8-PN formation by human gut microbiota, pooled fecal slurries of 12 donors were incubated with iXN as a substrate. After 24 h, 8-PN formation followed Michaelis-Menten kinetics with a V_max_ of 0.0005 µmol/h/g and a K_m_ of 30.59 µM, corresponding to a catalytic efficiency (k_cat_) of 0.016 mL/h/g feces, calculated as $\:{V}_{max}/{K}_{m}\:$(Fig. 2). These values were used as a benchmark for the average microbial 8-PN formation.

### Inter-individual differences in 8-PN formation

**Fig. 3:**
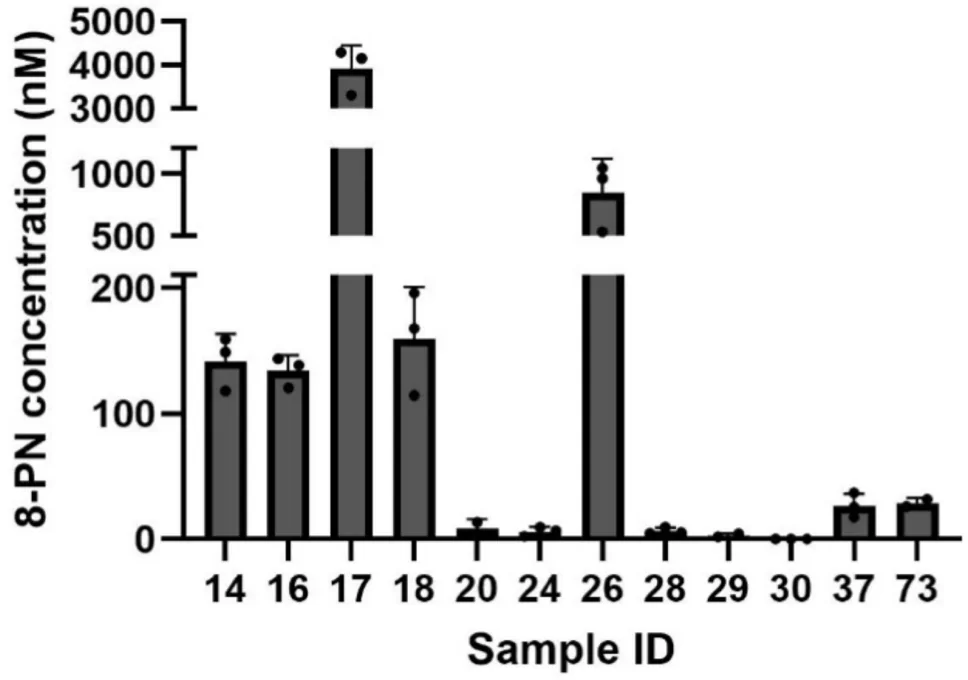
Interindividual variation in the 8-PN formation after anaerobic incubation of individual human stool samples with 10 µM iXN for 24 h (n = 12). Data are presented as mean ± SD of three replicates

Inter-individual variability in the microbial conversion of iXN to 8-PN was assessed using fecal slurries from 12 individuals. Anaerobic incubations were performed with a single concentration of iXN, or with DMSO (0.1%) as a solvent control. Among the 12 donors, one fecal microbiome (sample ID 30) showed no detectable iXN to 8-PN conversion (Fig. 3). Notably, the highest amount of 8-PN formed was observed in sample ID 17, followed by ID 26, with ID 17 producing ~ 600-fold higher levels than most other samples. Control samples confirmed the absence of background iXN and 8-PN, excluding contributions from prior dietary intake of hops-containing products. Donor-specific V_max_ values were extrapolated by scaling the pooled slurry V_max_ with relative 8-PN formation in each individual sample.

### PBK model-based predictions of 8-PN systemic and tissue levels

To account for the gut microbial formation of iXN to 8-PN in prediction of biologically relevant concentrations, we extended a recently published PBK model (Stevanoska et al. 2025) for predicting systemic and tissue concentrations of iXN and 8-PN. Applying the newly expanded model, we evaluated scenarios of taking a hop dietary supplement or one beer a day for seven consecutive days and predicted C_max_ in blood and uterus as organs of toxicological relevance for xenoestrogen activity. Additionally, since iXN and 8-PN co-occur in beer, the PBK model was applied to estimate their uterine ratios following consumption of low versus high-hopped beer (Supplementary information Table 1). Predicted uterine C_max_ values ranged from 0.02 to 0.15 nM for iXN and from 0.0004 to 0.002 nM for 8-PN, depending on hop polyphenol content. The corresponding 8-PN:iXN ratios averaged 1:100 in highly hopped beers and 1:50 in low-hopped beers. These ratios were subsequently tested in the ALP assay in Ishikawa cells to assess potential mixture effects on estrogenic activity.

**Fig. 4:**
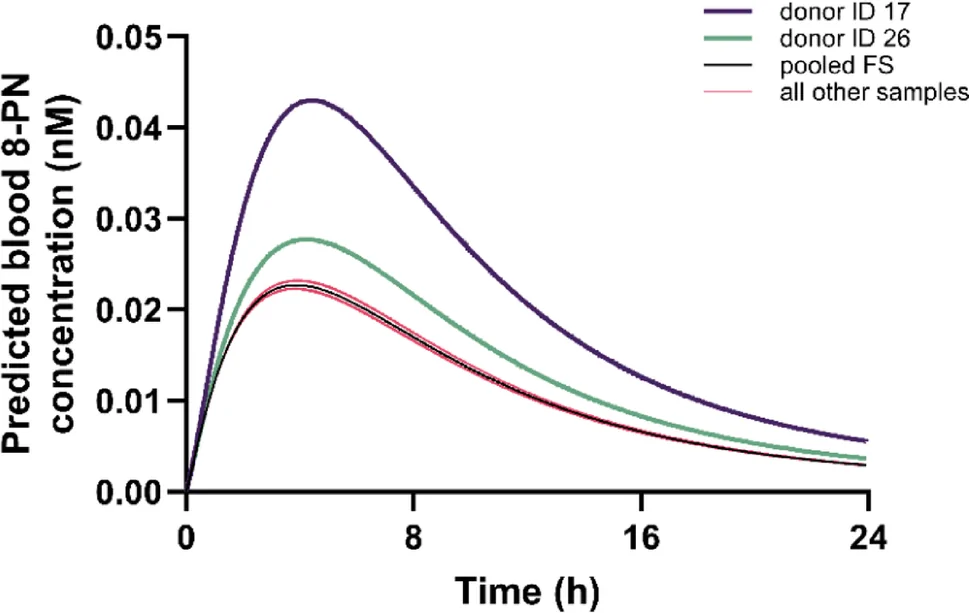
Predicted blood concentrations of 8-PN following supplementation with a hop supplement containing 3.2 mg iXN and 1 mg 8-PN. Simulations were performed using either the pooled fecal slurry V_max_ or the estimated donor-specific V_max_ values. Colored lines indicate individual predictions, and the black line denotes the pooled fecal slurry prediction

To investigate the role of interindividual variability in 8-PN formation, the PBK model was applied using donor-specific formation rates from 12 donors to predict blood concentrations of 8-PN following intake of a hop supplement (3.2 mg iXN and 1 mg 8-PN). The two highest metabolizers (IDs 17 and 26) were predicted to have markedly elevated 8-PN blood concentrations compared to all other donors (Fig. 4), with sample ID 17 exhibiting more than a two-fold higher level relative to the prediction based on the pooled fecal slurry.

**Fig. 5:**
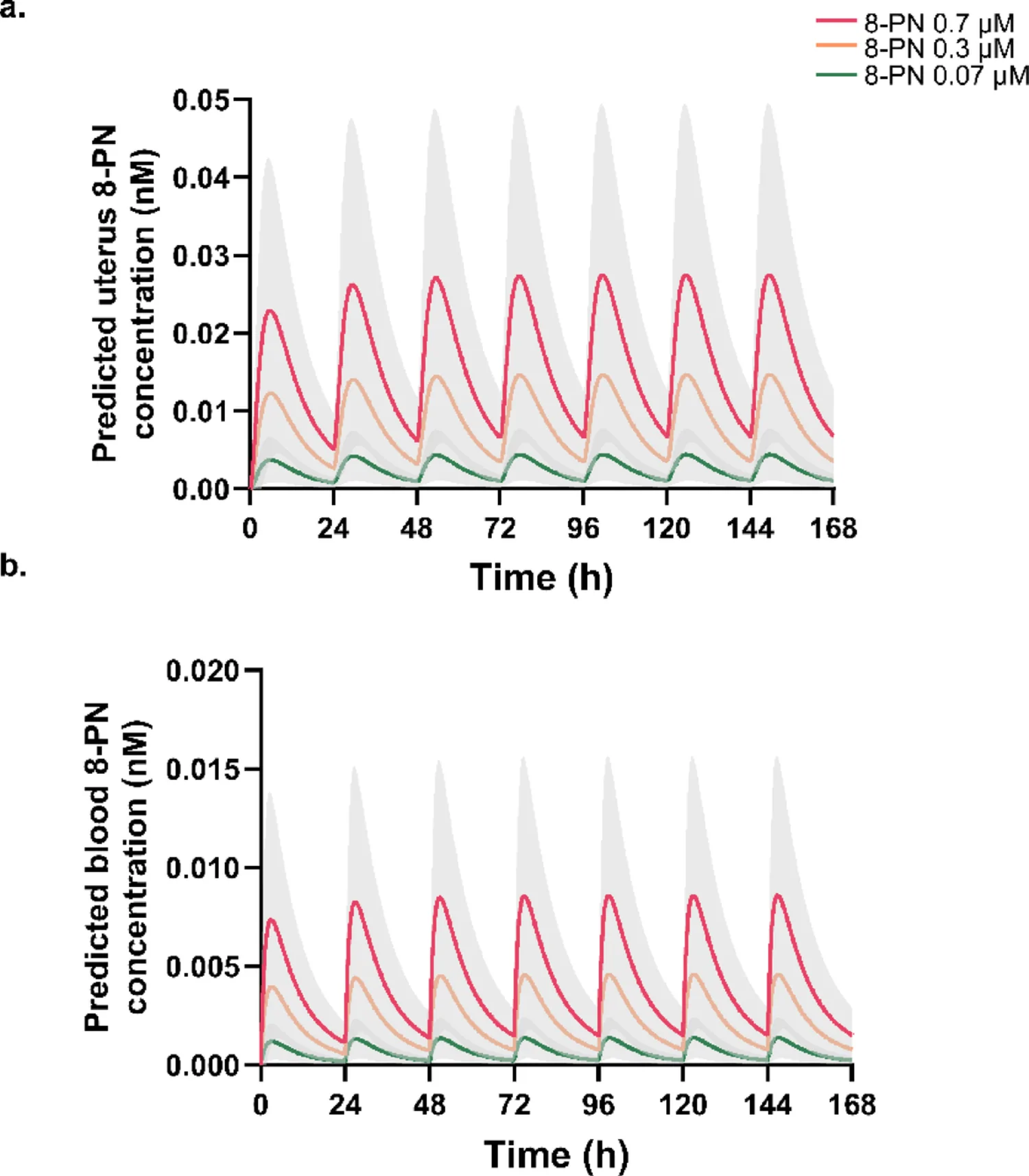
Predicted uterus (**a**) and blood (**b**) 8-PN concentrations following daily administration of three different beers for 7 days. Solid lines represent model predictions; shaded areas indicate Monte Carlo simulation outcomes (*n* = 1000, mean ± SD)

To account for population-based variation in blood and uterus 8-PN concentration, Monte Carlo simulations of 1000 unique input sets were used to simulate varied outcomes for three different beers with different concentrations of hop polyphenols consumed once daily for 7 days. The simulations revealed substantial variability, with some simulations showing a twofold increase in 8-PN concentration (Fig. 5). As the gut microbial formation of 8-PN was not a sensitive parameter, it was not used in the Monte Carlo simulations.

**Fig. 6:**
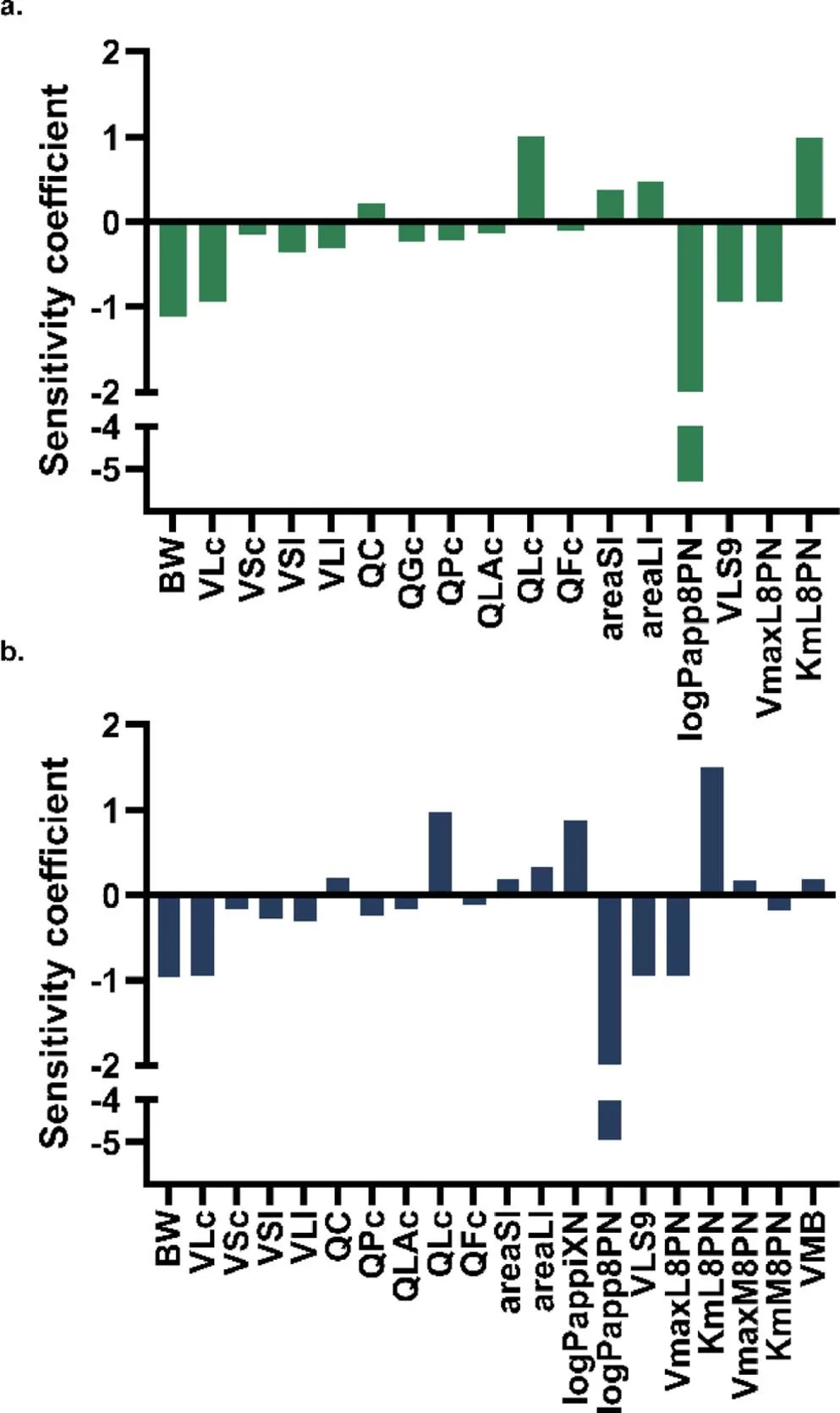
Sensitivity analysis of model parameters (**a** pooled V_max_, **b** donor-specific V_max_ of the highest 8-PN producer). Normalized sensitivity coefficients (SC) were calculated as changes of blood Cmax resulting from 5% increase in each parameter. Parameters with │SC│> 0.1 are shown; BW, bodyweight; VLc, relative liver volume; VSc, relative volume of slowly perfused tissues; VSI, small intestinal lumen volume; VLI, large intestinal lumen volume; QC, cardiac output; QGc, fraction of blood flow to gut; QPc fraction of blood flow to portal vein-perfused tissues; QLAc, fraction of arterial blood flow to liver; QLc, total fraction of blood flow to liver; QFc, fraction of blood flow to fat; areaSI, small intestinal lumen surface area; areaLI, large intestinal lumen surface area; logPappIXN, permeability coefficient of iXN across the intestinal barrier; logPapp8PN, permeability coefficient of 8-PN across the intestinal barrier of 8-PN; VLS9, scaling factor for S9 protein to liver tissue; VmaxL8PN, maximal velocity of hepatic 8-PN glucuronidation; KmL8PN, affinity constant for hepatic 8-PN glucuronidation VmaxM8PN, maximal velocity of microbial 8-PN formation; KmM8PN, affinity constant for microbial 8-PN formation; VMB, scaling factor for gastrointestinal tract contents

To evaluate the sensitivity of all individual parameters of the model, sensitivity analysis was performed (Fig. 6) using the V_max_ derived from pooled fecal slurry ex vivo fermentations and the donor-specific V_max_ for the highest 8-PN producer (ID 17). In both cases, C_max_ in blood was predominantly influenced by logP_app_ values of iXN and 8-PN, body weight, and fraction of blood flow directed to the liver. Primary differences between average vs. high 8-PN producers were observed in parameters related to metabolism. Unlike microbial kinetic parameters derived from the pooled slurry, those from the highest producer showed a sensitivity coefficient slightly above the threshold of 0.1.

### Predicted 8-PN blood concentrations in the presence and absence of CYP1A2-catalyzed iXN to 8-PN formation

**Fig. 7:**
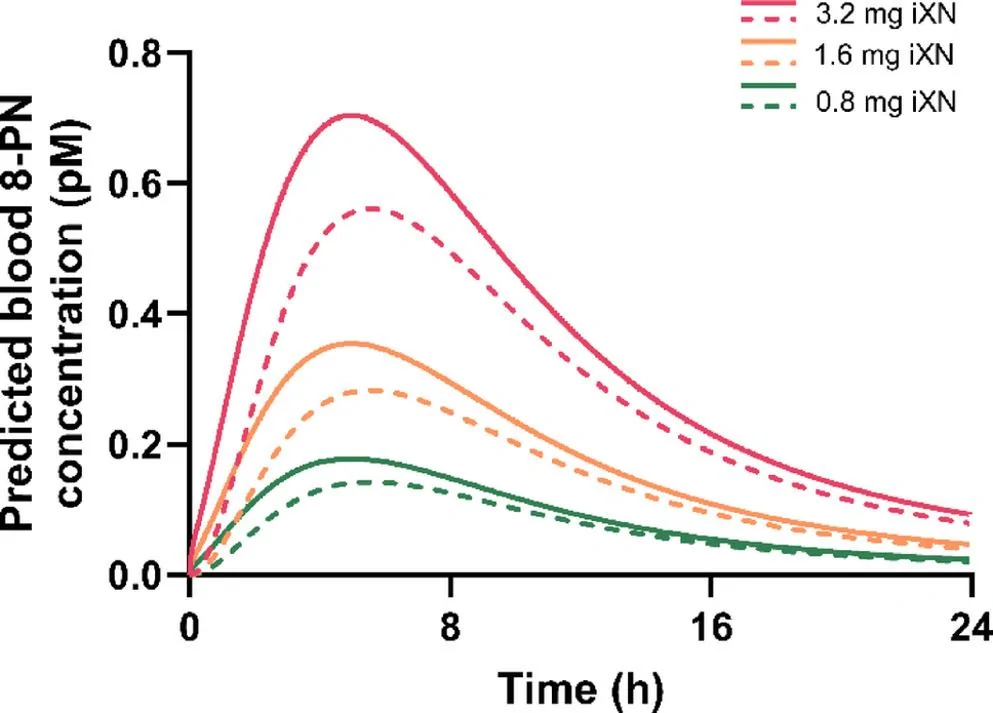
Predicted blood concentrations of 8-PN following three oral doses of iXN. Solid lines represent combined hepatic CYP1A2- and microbiome-mediated conversion of iXN to 8-PN, while dashed lines depict simulations excluding CYP1A2-mediated conversion

To evaluate the contribution of hepatic CYP1A2-mediated conversion of iXN to 8-PN, the PBK model was used to simulate biokinetics both with and without the inclusion of CYP1A2 activity. This was performed using three different iXN input doses, representing scenarios in which iXN was not hepatically converted to 8-PN. In the absence of CYP1A2 activity, the predicted 8-PN concentrations reflect only the microbial metabolism of iXN. The results indicate that hepatic CYP1A2 contributes to a 20–30% increase in 8-PN formation, depending on the iXN dose administered (Fig. 7), suggesting that hepatic conversion is relevant to overall 8-PN formation.

### Estrogenicity of iXN and 8-PN as single compounds

To evaluate the estrogenic potential of 8-PN and iXN, ALP activity was used as an indicator of estrogenic activity. Both compounds elicited maximal ALP activity comparable to that induced by 1 nM E2, confirming their estrogenicity; however, although their efficacy was similar, their potencies differed. 8-PN caused a significant ALP induction at much lower concentrations, with an EC_50_ of 1.77 nM and a lowest observed effect level (LOEL) of 1 nM (Fig. 7). In contrast, the EC_50_ of iXN was 1045 nM, and its LOEL was 500 nM, indicating that 8-PN is more than 100-fold more potent than iXN.

**Fig. 8:**
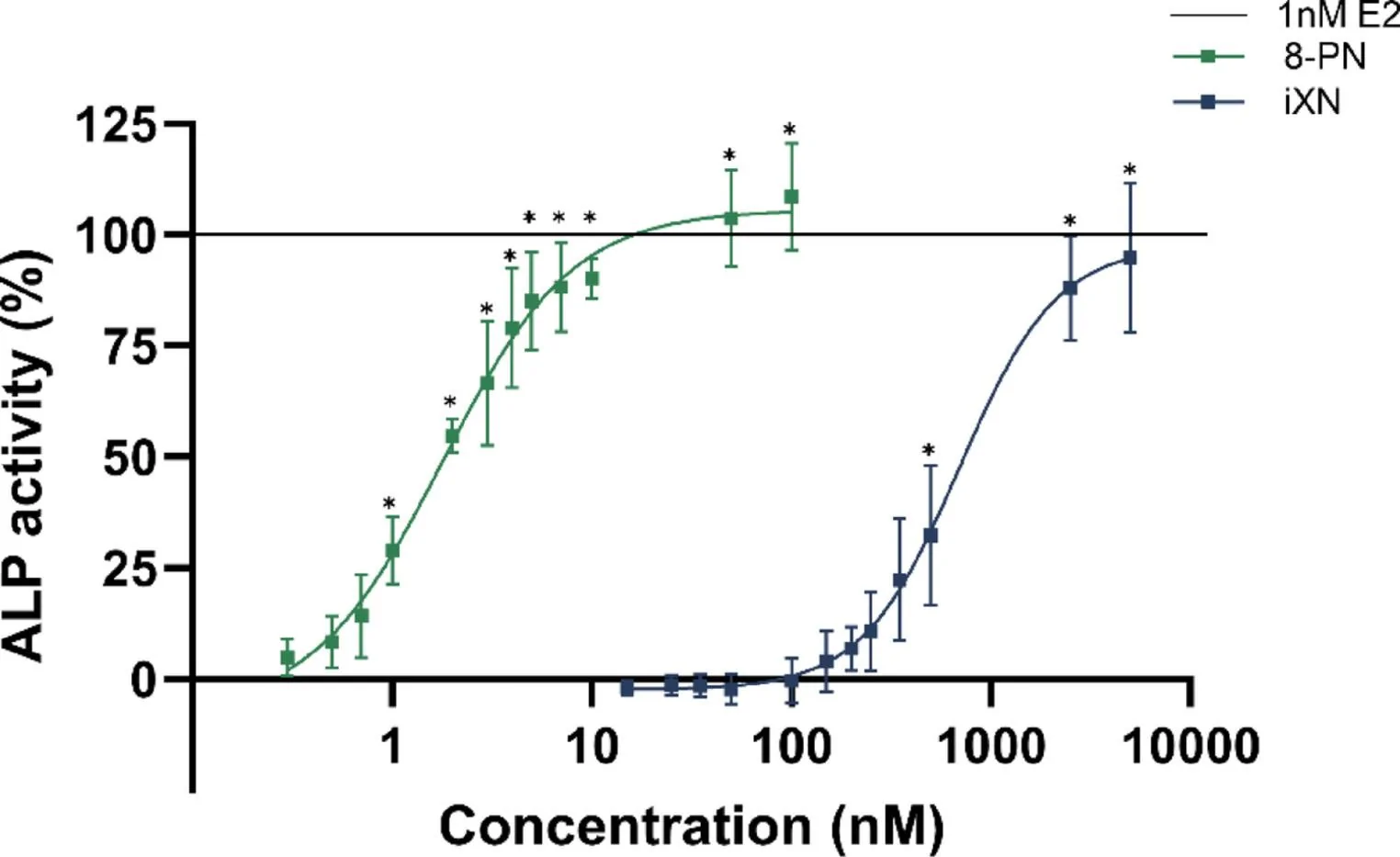
Induction of ALP activity by the hop polyphenols 8-PN and iXN. Values were scaled to the solvent control (0%; 0.1% DMSO) and the positive control (100%; 1 nM E2) and are presented as mean ± SD of ≥ 4 independent experiments. Significant differences from the no-effect level were determined by one-way ANOVA with Dunnett’s multiple comparisons indicated by * (*p* < 0.05)

### Estrogenicity of iXN and 8-PN mixtures

**Fig. 9:**
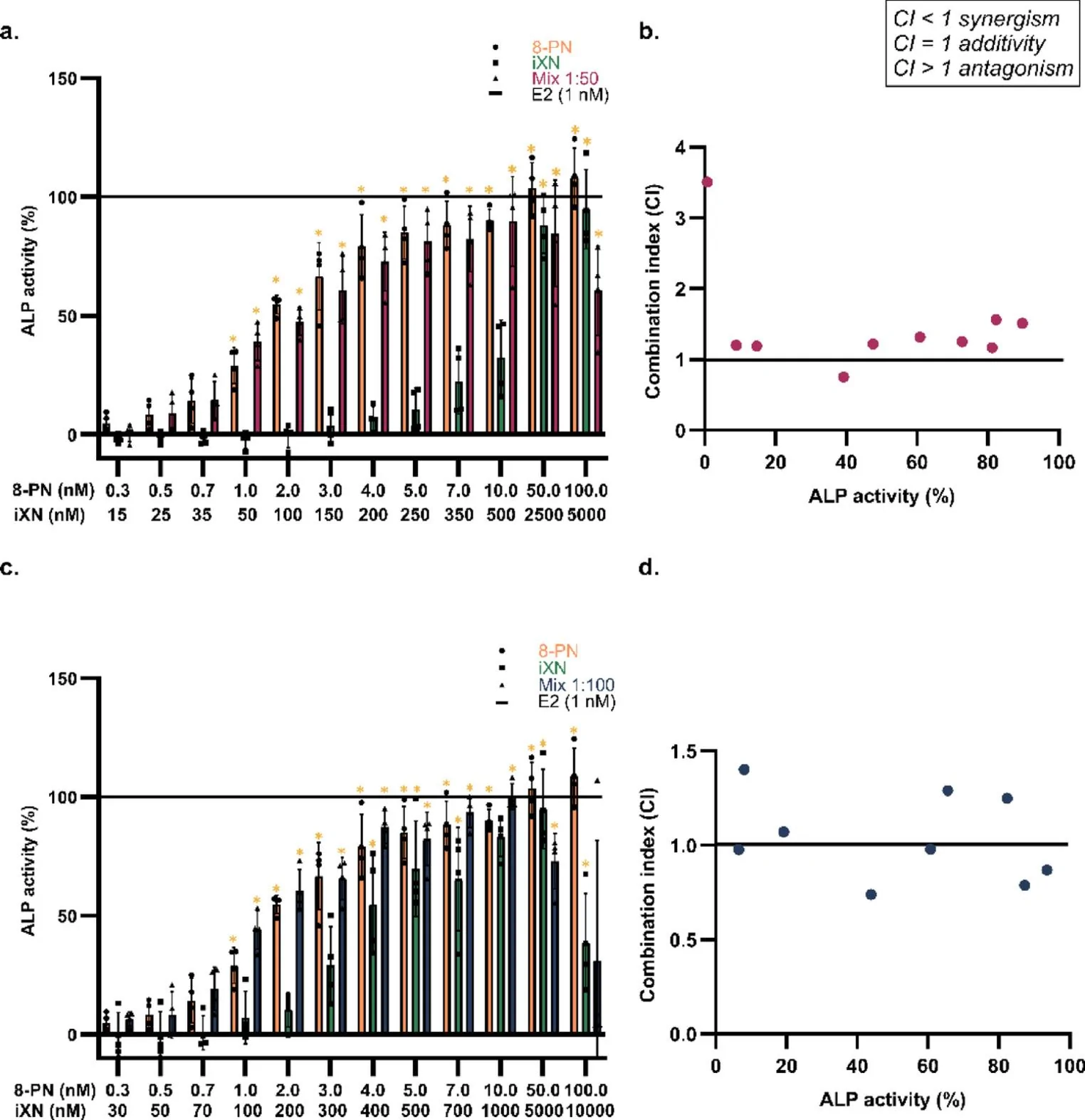
Estrogen receptor-dependent induction of ALP activity by iXN, 8-PN, or mixtures at 1:50 (**A**) and1:100 (**C**), normalized to the positive (1 nM E2) and negative control (0.1% DMSO). Data are presented as mean ± SD of four independent experiments. Graphs (**B**, **D**) show calculated combination index (CI)values based on the measured data. Significant differences from the no-effect level were determined by one-way ANOVA with Dunnett’s multiple comparisons indicated by * (*p* < 0.05)

To assess whether co-administration of iXN and 8-PN influences their estrogenic potential at physiologically relevant uterine concentrations, the microbiome-competent PBK model was utilized to predict 8-PN to iXN concentration ratios in the uterus under high and low-hopped beer dosing scenarios. These ratios (1:50 and 1:100, respectively) were selected to represent physiologically relevant conditions and to assess potential combinatory effects on estrogenicity in Ishikawa cells using the ALP assay, a well-established, license-free method based on ER-dependent expression of alkaline phosphatase as a natural reporter gene for estrogenic activity (Holinka et al. 1977). Concentration-dependent ER activation was determined for iXN and 8-PN alone (Fig. 8) and their combinations (Fig. 9). As thresholds of estrogenicity, in vitro point of departure (POD) concentrations were observed at 500 nM for iXN, 1 nM for 8-PN, and 1 nM 8-PN in both mixtures. To assess combinatory effects that potentially deviate from additive bioactivity, the Chou-Talalay method was used to calculate the combination index, allowing quantitative evaluation of their interactions (Chou 2006). For the 1:50 8-PN: iXN mixture, we observed varying interaction patterns across different ALP activity levels. Moderate synergism between 8-PN and iXN was observed at a concentration of 1 nM 8-PN with 39.2% ALP activity (CI = 0.751), indicating enhanced efficacy at these concentrations. At concentrations producing moderate ALP activity levels (e.g., 47.6–81.2%), the combination exhibited additivity or slight antagonism (1.165 ≤ CI ≤ 1.314). At higher activity levels (e.g., 89.7%), corresponding to 10 nM 8-PN and 500 nM iXN, the interaction shifted to antagonism (CI = 1.509). The highest concentrations of 8-PN and iXN lead to reduced estrogenicity as compared to the maximal effects. Similarly, CI values ranging from 0.7 to 1.4 were calculated for the estrogenicity of the 1:100 8-PN:iXN mixture. At moderate ALP induction levels, a slight synergism was observed, while for the mixture at a concentration of 4 nM 8-PN (65.6% ALP induction), slight antagonism was indicated. The observed antagonism at the lowest dose of the 1:50 mixture is likely due to variability at the lower detection limit of the assay. Across all activity levels and for both 8-PN to iXN ratios, the CI was relatively stable, indicating cumulative to slightly antagonistic effects. While the 1:50 ratio exhibited stronger antagonism at higher activity levels, the 1:100 ratio demonstrated more consistent additivity or mild synergism across the effect range.

## Discussion

By integrating ex vivo microbial kinetic data into a PBK model and linking it with in vitro estrogenicity testing, we characterized the contribution of the gut microbiome to internal exposure to the potent phytoestrogen 8-PN at target organs of endocrine disruption. The formation of 8-PN was experimentally quantified in both pooled and individual stool samples, revealing pronounced interindividual variability that was subsequently incorporated into the PBK model to capture microbiome-dependent differences in systemic exposure. Potential implications for consumer safety were evaluated by characterizing 8PN’s estrogenicity alone and in naturally occurring combination with its precursor iXN, using a well-established in vitro model.

To quantify 8-PN formation, ex vivo fecal fermentation experiments were conducted, and 8-PN was detected within 24 h, resembling average colonic transit times (Wang et al. 2015). The catalytic efficiency of 8-PN formation in pooled fecal samples (k_cat_ of 0.016 mL/h/g) was low compared with microbial biotransformation of other polyphenols; for example, the conversion of daidzein to S-equol was found to be more efficient with a k_cat_ of 1.24 mL/h/g (Wang et al. 2022). Consistent with this low catalytic rate, 8-PN was detectable only after 24 h of incubation. Integration of ex vivo kinetic parameters into the PBK model enabled the prediction of systemic and uterine 8-PN concentration under realistic intake scenarios. The model indicated that typical beer intake results in picomolar levels of 8-PN in blood and uterus, well below estrogenic effect thresholds (Aichinger et al. 2021; Milligan et al. 2002). While the model underwent internal checks (mass balance, unit consistency, sensitivity analysis) and prior evaluation against measured aglycone levels of iXN and 8-PN, external validation directly against parent 8-PN concentrations was not possible due to a lack of human pharmacokinetic data. Although typical beer consumption resulted in low picomolar concentrations of 8-PN, gut microbial interindividual variability can affect systemic 8-PN concentrations.

To investigate interindividual variability in biotransformation capacity, individual stool samples were incubated with a single concentration of iXN, revealing considerable variability in 8-PN formation among donors (Fig. 3). Of the twelve individual fecal slurries analyzed, only one produced no detectable 8-PN, whereas two exhibited remarkably high 8-PN levels reaching up to 3.9 µM within 24 h. Under identical conditions, pooled fecal samples yielded 298 nM, corresponding to a 13-fold difference between the average and the highest 8-PN producer. When donor-specific V_max_ values were implemented into the PBK model, predicted 8-PN blood concentrations were, as expected, markedly higher in strong producers with one donor (ID 17) predicted to experience higher concentration than the average exposure represented by pooled fecal kinetics (Fig. 3). These findings demonstrate interindividual variability in microbial 8-PN formation, emphasizing the microbiome’s contribution to variability in systemic exposure. The variability observed in these experiments reflects physiological metabolic capacity and aligns with previous classifications of microbiomes into poor, moderate, and strong 8-PN producers (Bolca et al. 2007; Possemiers et al. 2006). To explore microbial determinants of these interindividual differences, the composition of stool samples was profiled using 16 S rRNA amplicon sequencing. Relative abundances of bacterial communities at the phylum level and Shannon diversity indices were analyzed in relation to 8-PN production levels, and variance among donors was visualized by PCoA (Supplementary Figs. 1–3). Although E. *limosum* and E. *rammulus* have been associated with the microbial conversion of 8-PN (Moens et al. 2020; Paraiso et al. 2019), additional unidentified bacteria might play a role in the 8-PN formation. While 16s rRNA sequencing provided an overview of microbial composition, its limited resolution prevents precise identification of specific strains involved in 8-PN formation. The future application of metagenomic approaches could enable the identification of specific enzymes and corresponding genes responsible for 8-PN formation. Moreover, external factors such as lifestyle or age may also influence microbial 8-PN formation, and future analyses addressing these aspects could facilitate personalized risk assessment and targeted intervention strategies. As reviewed by Cortes-Martín et al. (2020), metabotype classification aids in understanding whether dietary polyphenols and their metabolites exert beneficial effects on the host. Establishing this concept for 8-PN is complex because the compound is present in trace amounts in dietary products, is not only produced by gut bacteria, but also in the human liver. Given this dual origin, it is necessary to consider hepatic metabolism as a contributing source of systemic 8-PN concentration.

Besides gut microbial enzymes, the hepatic enzyme CYP1A2 also converts iXN to 8-PN (Guo et al. 2006; Stevanoska et al. 2025) and it can substantially influence systemic levels of 8-PN. When we evaluated its impact on the systemic fate of iXN and 8-PN by simulation with or without CYP1A2-catalyzed conversion (Fig. 7), the model predicted CYP1A2 to induce a 30% increase in 8-PN blood concentration, i.e. a substantial hepatic contribution to systemic 8-PN levels for moderate microbial 8-PN producers. Moreover, CYP1A2 activity is modulated by genetic polymorphisms, such as the − 163 C > A variant, a single-nucleotide mutation in the gene’s promoter region that increases its expression and leads to higher metabolic activity, particularly in response to stressors such as cigarette smoke (Koonrungsesomboon et al. 2018). Consequently, individuals with the − 163 C > A CYP1A2 variant may produce elevated levels of 8-PN, also in combination with certain lifestyle factors, which may be further exacerbated if they are also a high microbial 8-PN metabolizer.

Considering 8-PN is a potent phytoestrogen, evaluating its endocrine activity is crucial, particularly for individuals with enhanced microbial and hepatic metabolism. A previous study suggested that iXN and 8-PN levels achieved through beer consumption induced estrogenic effects in Ishikawa cells (Aichinger et al. 2021). However, the concentrations of iXN and 8-PN that were tested were simply estimated based on dietary exposure. Therefore, in this study we characterized the estrogenicity of physiologically relevant 8-PN as well as a combination of 8-PN and iXN at realistic ratios. We focused on uterine concentrations due to the high expression of estrogen receptors in the uterus (Mylonas et al. 2004). For high and low-hopped beer intake scenarios, blood and uterine levels of 8-PN were predicted to be in the low nanomolar range, and there were slightly higher concentrations in the uterus. Additionally, 8-PN:iXN concentration ratios of 1:50 and 1:100 (Fig. 9) were tested as physiologically relevant combination exposure ratios in the uterus upon high- vs. low-hopped beer intake scenarios, respectively. The results indicated concentration-dependent interaction with synergistic effects at moderate ALP levels and additive or antagonistic effects at higher activity levels. Similar effects have been reported at a 1:500 8-PN:iXN ratio (Aichinger et al. 2021), suggesting dose-dependent interactions in ER transactivation. Importantly, the POD of the mixtures was not lower than the POD of 8-PN alone, indicating that combined effects are negligible for hazard assessment and that estrogenicity is primarily driven by 8-PN rather than iXN. Nevertheless, ALP responses can vary with cell culture and execution of assay protocols and should therefore be interpreted strictly within their experimental context. The assay serves as a useful preliminary screen, particularly given that predicted blood and target-tissue concentrations fall within the picomolar range. It should be noted that the POD determined in this study was derived using an estrogen-sensitive test system originating from uterine cells. While estrogenic endocrine-active substances can also affect other organ systems in both females and males, these effects are likewise considered to be mediated through interactions with estrogen receptors. Therefore, the POD derived in this study can be considered applicable to the majority of organ systems responsive to estrogens. However, it remains to be established whether this also holds true for individuals in estrogen-sensitive states, such as during specific developmental stages or in those with estrogen-sensitive breast cancer.

The results of this study suggest that there may be substantial interindividual variability, on the order of up to 600-fold, in the microbial conversion of iXN to 8-PN. However, significant estrogenic activity for 8-PN was observed at 1 nM, while even in high microbial producers, the predicted uterine 8-PN concentrations were at most 0.03 nM, which is below these POD values. These data suggest that typical dietary exposure to iXN and 8-PN, such as from drinking one beer each day, is unlikely to elicit estrogenic effects. Nevertheless, episodes of excessive consumption of highly hopped beer, also superimposed on high production due to individual microbiome factors and/or hepatic enzyme polymorphism, could lead to higher physiological concentrations of 8-PN. Finally, these findings highlight how interindividual variability in tissue-relevant chemical exposures can occur due to gut microbial bioactivation. While the consequences appear limited for 8-PN, such variability may be consequential for other microbial metabolites, particularly pharmaceuticals undergoing microbial metabolism, underscoring the utility of microbiome-competent PBK models to improve chemical safety assessment.

## Supplementary Information

Below is the link to the electronic supplementary material.

Supplementary Material 1

Supplementary Material 2

## Data Availability

The datasets generated and/or analyzed during the current study are available from the corresponding author on request.

## Code availability

The underlying PBK model code is available in supplementary information. To execute the code, copy and paste it into Berkeley Madonna.
