# Supplementary material for: Interindividual variability in gut microbial formation of the hop phytoestrogen 8-prenylnaringenin results in elevated but sub-toxic internal exposures
Source: Arch Toxicol. 2026 Mar 10;100(6):2397–409. doi: 10.1007/s00204-026-04329-8 (PMC13658542; doi:10.1007/s00204-026-04329-8)
Supplement: Supplementary file 1 [file 204_2026_4329_MOESM1_ESM.docx]

**Interindividual variability in gut microbial formation of the hop phytoestrogen 8-prenylnaringenin results in elevated but sub-toxic internal exposures**

Maja Stevanoska^1^, Michelle Cremona^1^, Karsten Beekmann^2^, Shana J. Sturla^1^ and Georg Aichinger^1,^*

Author affiliations:

^1^Department of Health Sciences and Technology, ETH Zurich, Switzerland

^2^Wageningen Food Safety Research (WFSR), Netherlands

*Corresponding Author:
Dr. Georg Aichinger
Laboratory of Toxicology, ETH Zurich
Schmelzbergstrasse 9, LFO D15.2
8092 Zurich, Switzerland
[georg.aichinger@hest.ethz.ch](mailto:georg.aichinger@hest.ethz.ch)

**16s rRNA data analysis**

Aliquots of fecal samples were sent to Microsynth AG (Balgach, Switzerland), where DNA was extracted and the V4 region of 16s rRNA genes was amplified and sequenced. The analysis was conducted with QIIME2 (version 2024.5). Cutadapt was used to remove the forward and reverse primers. Denoising and chimera filtering were performed with the DADA2 algorithm, and the reads were truncated at base 226 for forward reads and at 182 for reverse reads. The pretrained Greengenes2 20240926 classifier from the 515F/806R region was used to assign the taxonomy.

Figure 1. Principal coordinate analysis based on Bray-Curtis dissimilarity matrix to evaluate differences in community composition. Samples were color-coded based on two different groups of high and low 8-PN producers.

Figure 2. Relative abundance of bacterial taxa at the phylum level across all samples. Barplots represent the taxonomic composition of gut microbiota, based on 16S rRNA gene sequencing and classification using the Greengene database. Each bar corresponds to an individual sample, and colors indicate different bacterial phyla. Only taxa with a relative abundance ≥1% in at least one sample are shown; all others are grouped under “Others.”

Figure 3. Shannon diversity index of gut microbiome in 8-PN high and low producers. Alpha diversity was assessed using the Shannon entropy metric. Boxplots display the distribution of Shannon diversity values for each group (high: n = 5; low: n = 7), with individual samples overlaid as black dots.

*Table 1.* MacFarlane media composition

| **Ingredient** | **Amount for 1 L** | **Vendor** |
| --- | --- | --- |
| Cellobiose | 1.0 g | Alfa Aesar |
| Xylan (oat spelts) | 1.0 g | Apollo |
| Arabinogalactan (larch wood) | 1.0 g | Sigma |
| Inuline (Raftiline® HP) | 0.5 g | Sigma |
| Soluble starch (potato) | 1.0 g | Sigma |
| Amicase | 3.0 g | Sigma |
| Bacto™ Tryptone | 5.0 g | Thermo Scientific |
| Meat extract | 1.5 g | Sigma |
| Yeast extract | 4.5 g | Sigma |
| Mucine | 4.0 g | Sigma |
| Bile salt | 0.4 g | Sigma |
| KH_2_PO_4_ | 3.0 g | Sigma |
| NaHCO_3_ | 9.0 g | Sigma |
| NaCl | 4.5 g | Sigma |
| KCl | 4.5 g | Sigma |
| Mineral solution | 200 mL | see below |
| Tween 80 | 1.0 mL | Sigma |
| Volatile fatty acid mix | 5.7 mL | see below |
| 0.05 g/mL hemin | 1.0 mL | see below |
| ddH_2_O | 792 mL | / |
| Vitamin solution | 1.0 mL | see below |
| L-cysteine | 1.0 g | Sigma |

**Mineral solution**

| **Components** | **g/L** | **Vendor** |
| --- | --- | --- |
| MgSO_4_ anhydrous. (M: 120.37) | 3.05 g | Sigma |
| CaCl_2_∙2H_2_O (M: 147.02) | 0.5 g | Labguard |
| MnCl_2_∙4H_2_O (M: 197.91) | 1.0 g | Across Organics |
| FeSO_4_∙7H_2_O (M: 278.02) | 0.025 g | Sigma |
| ZnSO_4_∙7H_2_O (M: 287.56) | 0.5 g | Sigma |
| ddH_2_O | 1 L | / |

**0.05 g/mL Hemin stock solution**

| **Components** | **g/25 mL** | **Vendor** |
| --- | --- | --- |
| Hemin | 1.25 g | Across Organics |
| 3M NaOH | 5 ml | Fisher Chemicals |
| Milli-Q | 20 ml |  |

**Volatile fatty acid mix**

| **Components** | **For 100 mL** | **Vendor** |
| --- | --- | --- |
| Acetic acid | 32.8 mL | Merck |
| Propionic acid | 11.6 mL | Sigma |
| Isovaleric acid | 2 mL | Across Organics |
| Isobutyric acid | 1.6 mL | Across Organics |
| Valeric acid | 1.8 mL | Thermo Scientific |
| 5N NaOH | 50 mL | Fisher Chemicals |

**10x concentrated vitamin solution**

| **Components** | **g/100mL** | **Vendor** |
| --- | --- | --- |
| Biotin | 10 mg | Sigma |
| Cobalamin | 10 mg | Sigma |
| *p*-Aminobenzoic acid (PABA) | 30 mg | Sigma |
| Folic acid | 50 mg | Sigma |
| Pyridoxamine | 150 mg | Sigma |
| ddH_2_O | Fill up to 100 mL | / |

Table 2. Concentrations of isoxanthohumol and 8-prenylnaringenin in beer, quantified by UHLPC-MS/MS based on data reported by Golubovic et al. (2021).

| ***Highly hopped beers*** | **Isoxanthohumol (µM)** | **8-prenylnaringenin (µM)** |
| --- | --- | --- |
| H2 | 3.979 | 0.068 |
| H13 | 4.148 | 0.063 |
| H15 | 3.64 | 0.042 |
| ***Low hopped beers*** |  |  |
| H26 | 0.553 | 0.015 |
| H3 | 1.56 | 0.051 |
| H19 | 1.608 | 0.034 |

*Table 3.* PBK parameters used for the Monte Carlo simulations

| ***PBK parameter description*** | ***Variable name*** | ***Sample distribution*** |
| --- | --- | --- |
| Body weight | BW | Normal |
| Relative liver tissue volume | VLc | Normal |
| Relative volume of all other organs | VRestc | Normal |
| Volume of SI | VSI | Normal |
| Volume of LI | VLI | Normal |
| Cardiac output | QC | Normal |
| Blood flow to gut tissue | QGc | Normal |
| Blood flow to portal vein perfused tissues | QPc | Normal |
| Blood flow to liver via artery | QLAc | Normal |
| Total blood flow to liver | QLc | Normal |
| Blood flow to slowly perfused tissues | QSc | Normal |
| Blood flow to adipose tissue | QFc | Normal |
| Partition coefficient rapidly perfused tissues | PR8PN | Log-normal |
| Surface area SI | areaSI | Normal |
| Surface area LI | areaLI | Normal |
| Permeability coefficient 8-PN | logPapp8PN | Normal |
| Scaling factor (mg S9 protein/gram liver) | VLS9 | Normal |
| Maximum rate | VmaxL8PN | Log-normal |
| Affinity constant | KmL8PN | Log-normal |
